# Fabrication and characterization of a smart film based on cassava starch and pomegranate peel powder for monitoring lamb meat freshness

**DOI:** 10.1002/fsn3.2918

**Published:** 2022-05-13

**Authors:** Azadeh Esfahani, Abdorreza Mohammadi Nafchi, Homa Baghaei, Leila Nouri

**Affiliations:** ^1^ 68106 Department of Food Science and Technology Damghan Branch Islamic Azad University Damghan Iran; ^2^ 26689 Food Technology Division School of Industrial Technology Universiti Sains Malaysia Penang Malaysia

**Keywords:** active packaging, antioxidant activity, cassava starch, intelligent film, pomegranate peel, TVB‐N

## Abstract

Nowadays, the development of pH‐sensitive smart edible films using biopolymers and natural plant extracts (especially those rich in anthocyanins) has attracted much attention. Therefore, in this study, the intelligent edible film was produced and characterized using cassava starch and pomegranate peel powder (PPP) and the possibility of using production films to monitor the freshness of lamb meat. The smart films were prepared using different concentrations of PPP (2, 4, 6, and 8% w/w) and the solvent casting method. The results showed that the incorporation of PPP had a significant effect on the mechanical parameters of the starch films. With increasing the levels of PPP, the color of the films became darker and redder. Increasing the PPP levels also led to an increase in total phenol content (TPC) (from 0 to 13 mg GAE (gallic acid equivalent)/g) and antioxidant activity (from 0% to 70% DPPH (1,1‐diphenyl‐2‐picryl hydrazyl) radical scavenging) of the produced films (*p* < .05). The intelligent film was used in the lamb meat packaging, and the color of the film changed from red to green during the storage period at 25°C. The amount of total volatile basic nitrogen (TVB‐N) in the meat could be detected by color changes of the intelligent films. Finally, this study demonstrated that the film based on cassava starch and PPP could be used as an intelligent and pH‐sensitive film to monitor the freshness of meat and meat products.

## Graphical abstract

An intelligent edible film was produced and characterized using cassava starch and pomegranate peel powder (PPP) and the possibility of using production films to monitor the freshness of lamb meat. The amount of total volatile basic nitrogen (TVB‐N) of the meat was measured, and a clear relationship was observed between the color change of the film and the TVB‐N value. This study demonstrated that the film based on cassava starch and PPP could be used as an intelligent and pH‐sensitive film to monitor the freshness of meat and meat products.

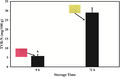

## 1 INTRODUCTION

Meat and meat products are rich sources of natural nutrients such as amino acids, vitamins, minerals, and high‐quality proteins and play an important role in the human diet (Yehia et al., 2021). However, these valuable products are in the category of highly perishable food products because they are nutritionally rich, contain unsaturated lipids in their composition, and have high water activity. Consequently, meats are sensitive to microbial growth and oxidative spoilage (Vergara et al., 2021). In the food industry, the common packaging systems are used to prevent contamination of food products as well as to protect them against diverse and unfavorable physicochemical conditions’ stresses (Chang et al., 2021; Han et al., 2018; Kalpana et al., 2019). In general, active packaging (containing active agents) has more functions than conventional packaging systems. It plays a more significant role in maintaining the quality and preservation of food products (Dodero et al., 2021).

One of the new and effective technologies in the food industry is intelligent biopolymer packaging, which is in the smart packaging category and provides the consumer with good information about the state of freshness or spoilage of food products, so these food packagings are receiving more and more attention (Azlim et al., 2022; Vedove et al., 2021; Xue Mei et al., 2020). Smart packaging films can display the freshness of food products, develop shelf life, and improve the quality and safety of food products during transportation and storage (Chen et al., 2020; Mahmood et al., 2022). One of the intelligent systems used to detect the freshness of food products is pH‐sensitive packaging, which consists of two parts, including a biopolymer film and a pH‐sensing active agent (especially natural pigments such as anthocyanin; Azlim et al., 2022; Taherkhani et al., 2020). A pH‐sensitive intelligent packaging has been successfully used to display the freshness of food products, especially meat and meat products (Alamdari et al., 2021; Alizadeh‐Sani et al., 2021; Ghorbani et al., 2021; Li et al., 2022; Zhou et al., 2021). Due to the microbial spoilage of meat products, biogenic amines including dimethylamine, trimethylamine, and ammonia are produced, which are also called total volatile basic nitrogen (TVB‐N) (Efremenko & Mirsky, 2017). Therefore, TVB‐N levels can be an effective indicator of meat freshness. The growth of microbes in meat will gradually increase the amount of TVB‐N. The product condition becomes more alkaline, and the pH change could be detected by pH‐sensitive films (Dong et al., 2020).

Anthocyanins are among the flavonoids’ secondary metabolites and water‐soluble pigments found in many vegetables and fruits. These known pigments have shown higher antioxidant activity than ascorbic acid and tocopherols and have shown color changes in response to pH change (Rawdkuen et al., 2020). When anthocyanins are exposed to environments at different pHs, they form other chemical structures, each with a different color. Therefore, these pigments can be effectively used to display the freshness of food products in biopolymer packaging films (Moradi et al., 2019).

Pomegranate (*Punica granatum*) is a world‐famous fruit rich in bioactive and functional compounds such as flavonoids, anthocyanins, and polyphenols. Remarkable biological activities have been reported for different parts of the pomegranate fruit, including anticarcinogenic, antiviral, antimicrobial, anti‐inflammatory, antioxidant, and antidiabetic activities (Chen, Liao, et al., 2020; Kyriakidou et al., 2021). Pomegranate peel makes up almost 26–30% of the fruit weight and is one of the important by‐products of juice factories, rich in various bioactive compounds (Sharma & Yadav, 2020; Spilmont et al., 2015). Researchers have approved that the content of bioactive compounds and functional activity of pomegranate peel are higher than those of its edible part (Masci et al., 2016). Pomegranate peel has successfully improved food products’ quality and shelf life (Chen, Liao, et al., 2020; Sharma & Yadav, 2020). To the best of our knowledge, biopolymer films with pomegranate peel powder (PPP) have not been investigated as an intelligent indicator. Therefore, this study aimed to evaluate the antioxidant activity and mechanical properties of cassava starch‐based films containing PPP and the possibility of using this active and intelligent film as a freshness indicator for lamb meat.

## 2 MATERIALS AND METHODS

### 2.1 Materials

Cassava starch was purchased from SIM Supply Company (Penang, Malaysia). Pomegranate fruit (Malas variety) was purchased from a local market (Saveh, Iran), and the peels were separated and washed. Then the peels were dried in the shade for 10 days and powdered by a laboratory‐scale mill. Glycerol as a plasticizer and other chemicals used in this research were purchased from Merck (Merck Millipore, Darmstadt, Germany).

### 2.2 Preparation of cassava starch/PPP films

To prepare the starch suspension, 3% w/v of cassava starch was dispersed in cold distilled water and stirred thoroughly at room temperature for 1 min to become uniform. Glycerol (as plasticizer) was added to the starch suspension at 30% w/v of starch and PPP at 2%, 4%, 6%, and 8% w/w. The resulting mixtures were heated on a magnetic stirrer for 25 min to form a homogeneous solution. The solutions (100 ml) were uniformly spread on a 300‐µm Teflon and dried at room temperature for 48 h (Chi et al., 2020). Then dried films were peeled off and stored in a desiccator (relative humidity (RH) = 50–55%, 25°C) in a dark place.

### 2.3 Evaluation of the mechanical properties of films

The mechanical properties of the starch‐based films, including tensile strength (MPa), Young's modulus of elasticity modulus (MPa), and elongation at break (%), were evaluated with a texture analyzer and according to the ASTM D882‐18 standard method with some modifications (Babapour et al., 2021). The samples were cut to dimensions of 100 mm × 20 mm and conditioned at 23°C and 53% relative humidity (RH) for 48 h. The initial distance of the two probes of the device was 50 mm, and the separation speed was 30 mm/min. The cell bar and the probe speed of the texture analyzer were 1 kN and 50 mm/min, respectively.

### 2.4 Evaluation of the total phenol content (TPC) of films

The total phenol content (TPC) of the starch films was evaluated using the Folin–Ciocalteu method. The film sample (50 g) was dissolved in acetic acid (10 ml), and the solution (100 µl) was mixed with Folin–Ciocalteu reagent (200 μl) and distilled water (2 ml). Sodium carbonate (Na_2_CO_3_) (20%, w/v) (1 ml) was then added to the mixture and kept for 25 min at 50°C, and finally, its absorbance was read using a spectrophotometer at 765 nm. The gallic acid standard curve was used for determining the TPC amounts of film samples, and TPC was expressed as milligrams of gallic acid equivalent (GAE)/g of the film (Kurek et al., 2021; Shortle et al., 2014).

### 2.5 Evaluation of the antioxidant activity of films

The antioxidant activity of the film samples was determined using the 1,1‐diphenyl‐2‐picryl hydrazyl (DPPH) free radical‐scavenging ability method (Kumarasamy et al., 2007; Qin et al., 2015). At first, to obtain a DPPH solution with 0.1 mmol/ml concentration, DPPH (4 mg) was dissolved in methanol (MeOH) (100 ml). After that, the film extract solution (2 ml) was mixed with the DPPH solution (2 ml). The mixture was then kept at room temperature and in a dark place for 30 min. Finally, the absorbance of sample solution (As) was read using a spectrophotometer at 517 nm against the blank (MeOH) (Ac). The inhibition of the DPPH‐free radical was calculated using Equation (1) and expressed as a percentage (%):

(1) $$\text{DPPH}\;\text{inhibition}\left(\%\right)=\frac{1‐\text{As}}{\text{Ac}}\times 100$$

### 2.6 Determination of the color of films

The color parameters studied in this research included L* (lightness), a* (redness/greenness), and b* (yellowness/blueness), which were examined by a colorimeter (Minolta CM‐3500d; Minolta Co. Ltd., Osaka, Japan; Abedinia et al., 2018). The total color change (Δ*E*) of the films was also calculated using Equation (2):

(2) $$\Delta E=\sqrt{{\left(L_{0}‐L_{t}\right)}^{2}+{\left(a_{0}‐a_{t}\right)}^{2}+{\left(b_{0}‐b_{t}\right)}^{2}}$$

### 2.7 Study of pH‐sensitive action of film for monitoring the freshness of lamb meat

In this study, the method expressed by Chi et al. (2020) was used to monitor lamb meat's freshness. The lean meat of lamb without fat was first cut into pieces with a dimension of ~1 cm^3^. The film sample (10 mm × 10 mm) was then fixed on the internal surface of the Petri dish headspace containing 30 g of lamb meat at 25°C. When the color film changed to green, the amount of total TVB‐N in meat was measured.

To determine the TVB‐N content of meat, 10 g of meat sample and 2 g of magnesium oxide were evenly dispersed in 300 ml of distilled water. The solution was then diluted in 50 ml of boric acid, and methyl red reagent was added to it, and after that it reached a volume of 150 ml. The solution was titrated with hydrochloric acid (HCl). Finally, the amount of TVB‐N of the sample was calculated by Equation (3) and reported as mg/100 g of meat, where V is the volume of HCl added and C is its concentration:

(3) $$\text{TVB}‐N\left(\frac{\text{mg}}{100g}\right)=\frac{V\times C\times 14\times 100}{10}$$

### 2.8 Statistical analysis

Statistical analysis of data obtained from the tests was done using IBM SPSS Statistics 22.0 (USA). One‐way analysis of variance (ANOVA) followed by Duncan's multirange post hoc test was used to compare means at *p* < .05 significance level among different samples.

## 3 RESULTS AND DISCUSSION

### 3.1 Mechanical properties of cassava starch films containing PPP

**FIGURE 1:**
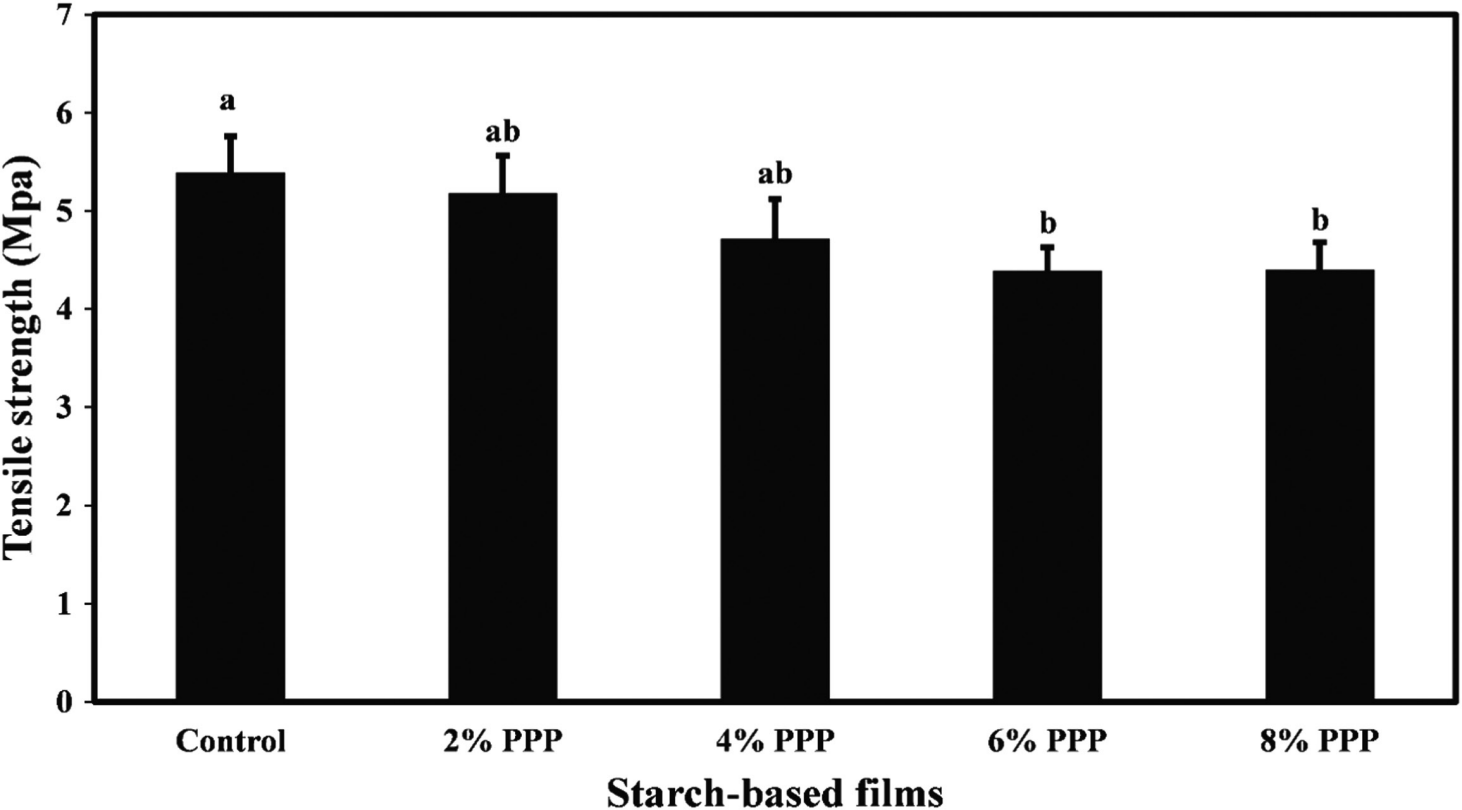
Comparison of the tensile strength (MPa) mean values of cassava starch‐based films containing different levels of pomegranate peel powder. Bars represent mean (*n* = 3) ± *standard deviation*. Different letters on the bars indicate a significant difference at 5% level of probability among film samples. PPP: pomegranate peel powder

**FIGURE 2:**
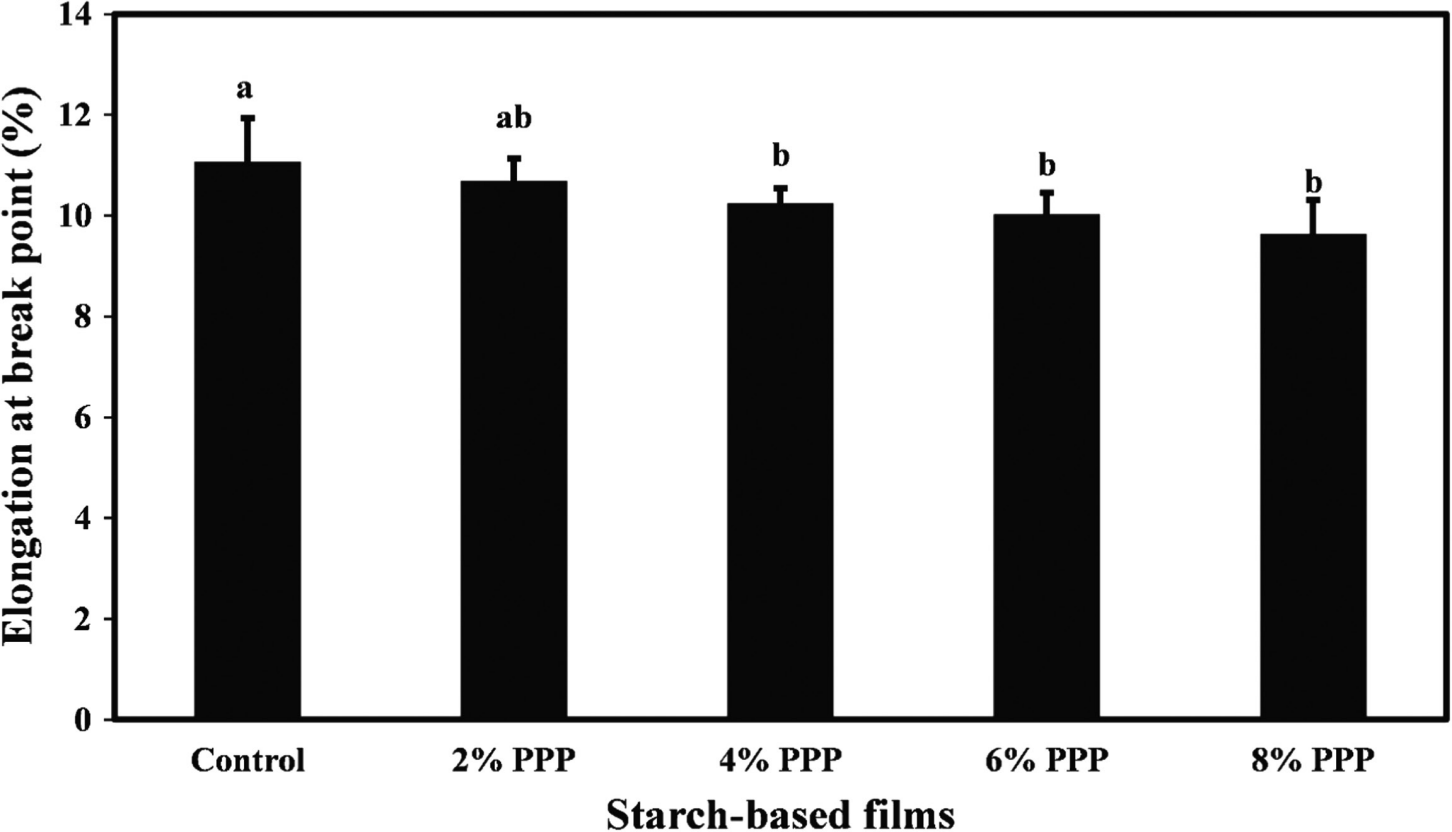
Comparison of Young's modulus (MPa) mean values of cassava starch‐based films containing different levels of pomegranate peel powder. Bars represent mean (*n* = 3) ± *standard deviation*. Different letters on the bars indicate a significant difference at 5% level of probability among film samples. PPP: pomegranate peel powder

**FIGURE 3:**
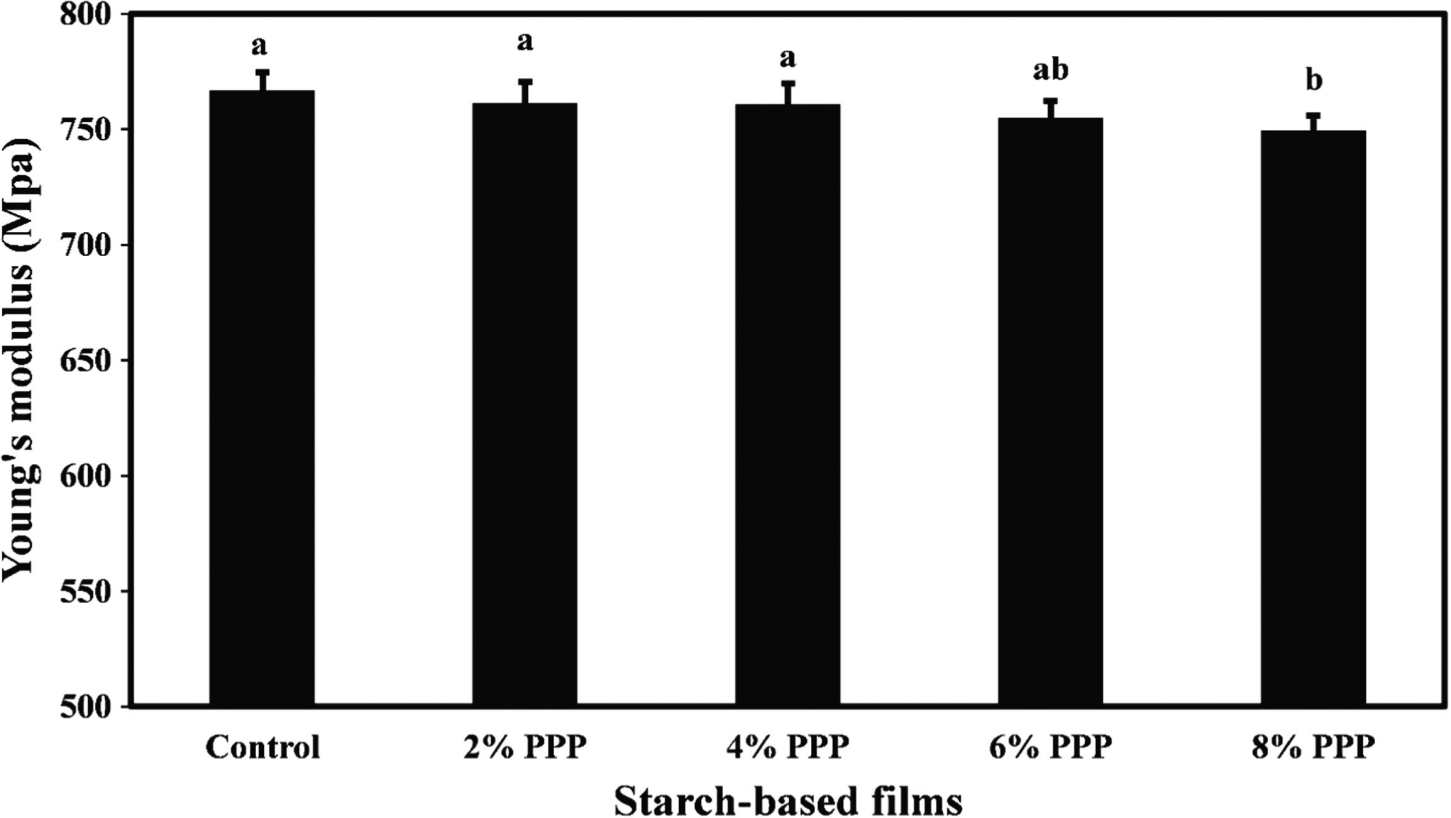
Comparison of elongation at break point (%) mean values of cassava starch‐based films containing different levels of pomegranate peel powder. Bars represent mean (*n* = 3) ± *standard deviation*. Different letters on the bars indicate a significant difference at 5% level of probability among film samples. PPP: pomegranate peel powder

The mean values of tensile strength, Young's modulus, and elongation at the breaking point of cassava starch films containing different levels of PPP are compared in Figures 1, 2, 3, respectively. They show that the mean values of tensile strength, Young's modulus, and elongation percentage of the film samples were in the range of 4.40–5.39 MPa, 749.41–766.78 MPa, and 9.64–11.06%, respectively. Increasing the levels of PPP in the samples led to a decrease in the mechanical properties of the films; however, these changes were not statistically significant. A minor reduction in mechanical properties of biopolymer‐based films was observed. This is likely due to the incorporation of bioactive agents with functional compounds and a decrease in density of the film matrix structure, which reduces the percentage of elongation and mechanical strength of films (Averous et al., 2001). Adding bioactive agents to the film formulation can also increase the hydrophilicity of the films and help the films absorb more moisture, thereby reducing the strength of the films. In general, the mechanical strength of the films depends on various factors such as additive types, film thickness, fabrication method, test speed, and the program used for the tensile test (Veiga‐Santos et al., 2011).

Similarly, Yong et al. (2019) observed that incorporating 10% and 15% levels of purple sweet potato into chitosan‐based films reduced the tensile strength and elongation percentage of the film samples. Still, this reduction in the film strength was not statistically significant. Andretta et al. (2019) also stated that despite the decrease in tensile strength and Young's modulus and the increase in elongation percentage of cassava starch‐based films due to the incorporation of blueberry residue, these changes in the mechanical properties of films were not significant.

### 3.2 Effects of PPP on color parameters of cassava starch films

**FIGURE 4:**
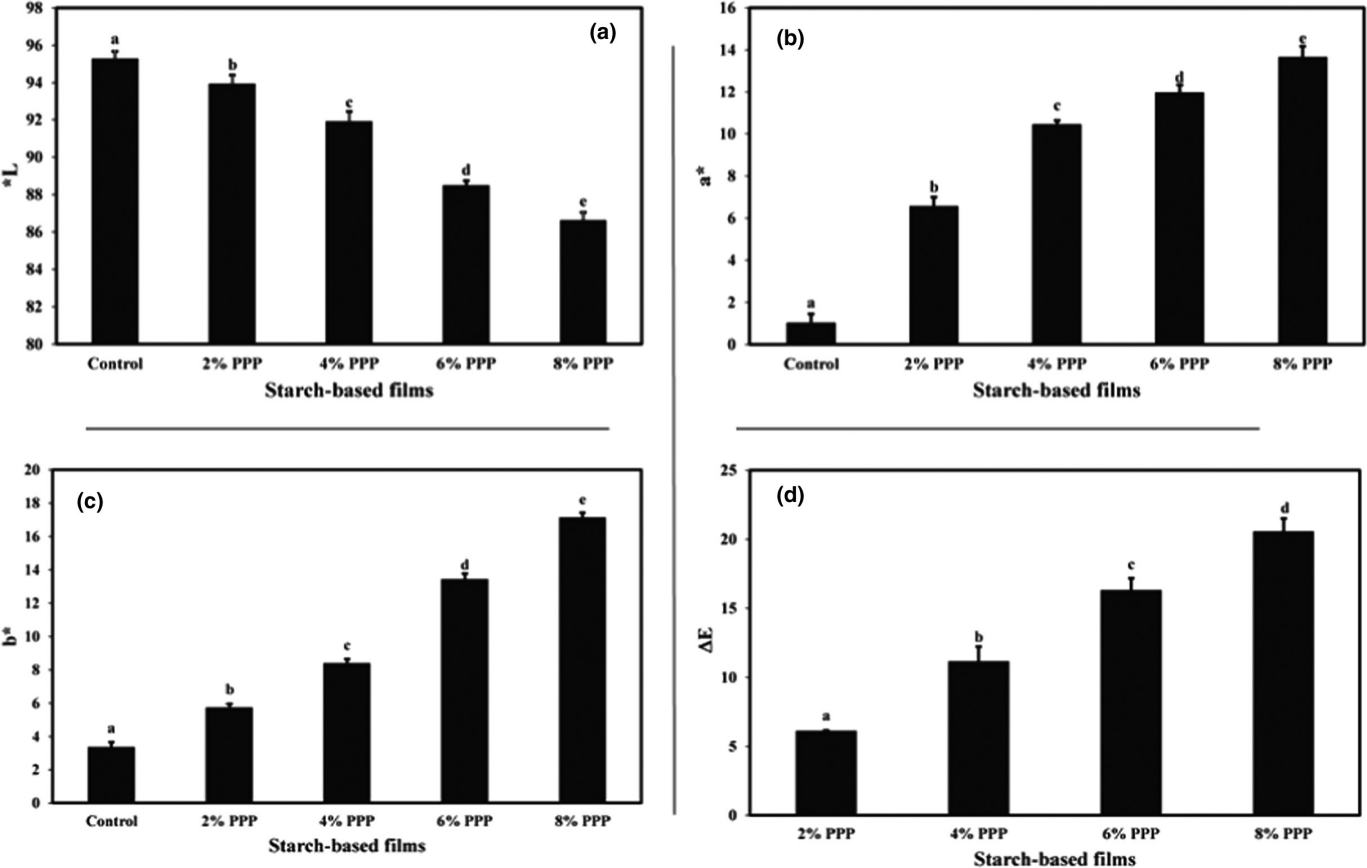
Comparison of (a) L*, (b) a*, (c) b*, and Δ*E* index mean values of cassava starch‐based films containing different levels of pomegranate peel powder. Bars represent mean (*n* = 3) ± *standard deviation*. Different letters on the bars indicate a significant difference at 5% level of probability among film samples. PPP: pomegranate peel powder

The appearance and color of films used for food packaging are major factors that significantly impact consumer satisfaction with packaging films (Ghasemlou et al., 2011). In Figure 4, the mean values of L*, a*, b*, and Δ*E* of the cassava starch films containing different levels of PPP are compared, respectively. As can be seen in the figures, the control film had the highest L* value (95.29) and the lowest values of a* (1.05) and b* (3.39). With increasing the level of PPP from 2% to 8% in starch‐based films, the lightness of the color significantly decreased. The redness and yellowness, as well as the total color change of the films, increased significantly (*p* < .05), so that the lowest value of L* (86.64) and the highest values of a* (13.67), b* (17.15), and Δ*E* (20.58) were observed in the films containing the highest level of PPP (8% level). In general, with increasing the levels of PPP in the cassava starch‐based films, the color of the film samples became darker.

Emam‐Djomeh et al. (2015), in their study of the effect of pomegranate peel extract on the color indexes of sodium caseinate‐based films, observed that the addition of this additive significantly reduced the lightness and increased the a*, b*, and Δ*E* indexes. Researchers also observed the darkening of the high‐amylose starch‐based films due to the addition of pomegranate peel (Ali et al., 2019).

### 3.3 Effects of PPP on the TPC of cassava starch films

**FIGURE 5:**
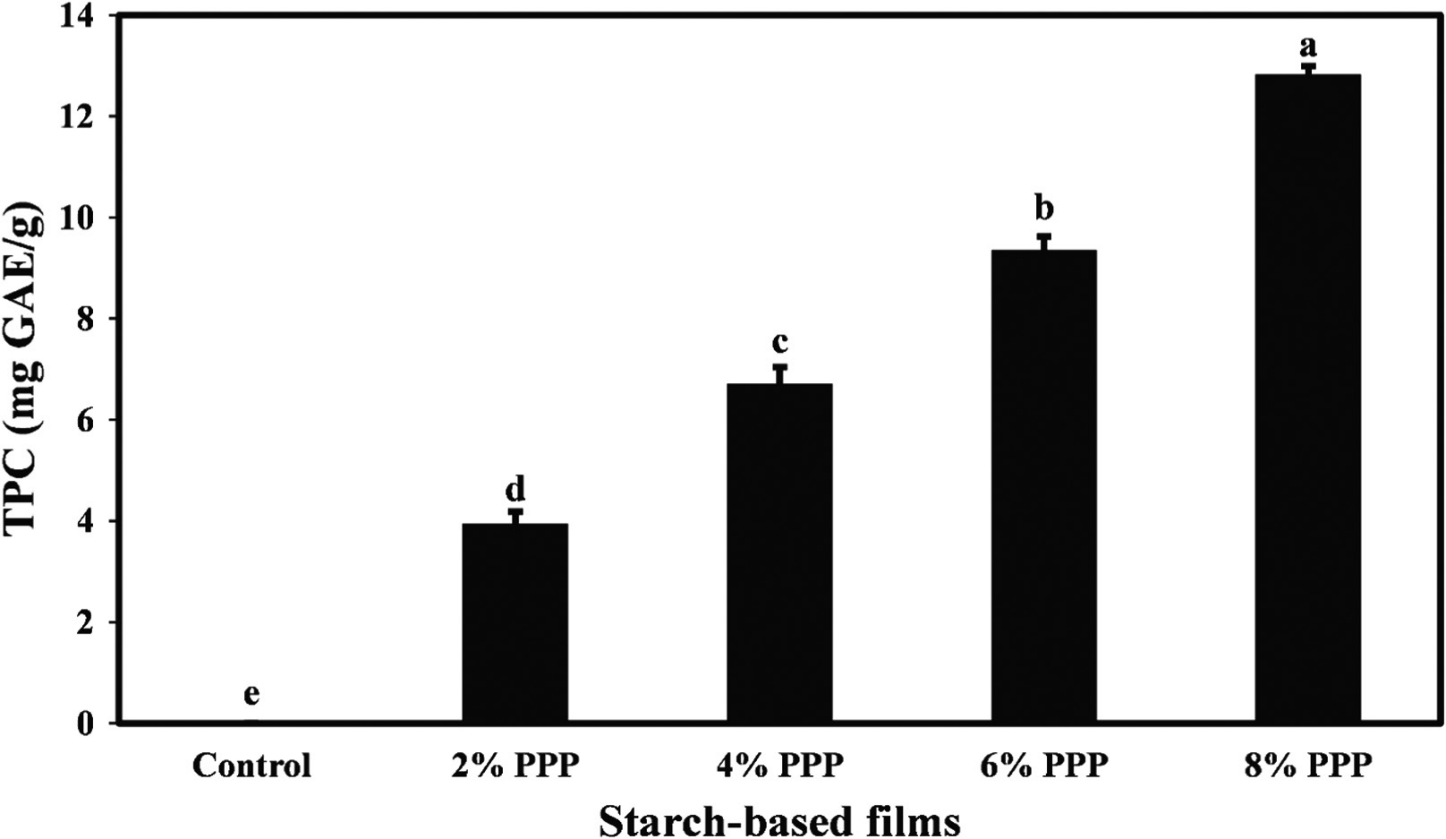
Comparison of TPC means values (mg GAE/g of the dried film) of cassava starch‐based films containing different levels of pomegranate peel powder. Bars represent mean (*n* = 3) ± *standard deviation*. Different letters on the bars indicate a significant difference at 5% level of probability among film samples. PPP: pomegranate peel powder. TPC: total phenol content. GAE: gallic acid equivalent

Phenolic compounds are naturally occurring plant secondary metabolites that exhibit major and key biological roles, especially radical scavenging and antioxidant activity (Sami et al., 2017). The values of TPC of cassava starch films containing different levels of PPP were measured by the Folin–Ciocalteu method, and the results are presented in Figure 5. As expected, the control film did not contain phenolic compounds. By adding PPP to the starch‐based films and increasing its concentration from 2% to 8%, the TPC of samples increased significantly (*p* < .05), so that the highest TPC was observed in the film containing 8% PPP (12.83 mg GAE/g of the dried film). Gallic acid, punicalagin, ellagic acid, catechin, quercetin, etc., are important polyphenol compounds in pomegranate peel (Akhtar et al., 2015).

A significant increase in TPC of chitosan‐based films was also observed due to the incorporation of pomegranate peel extract (Fan et al., 2013). Other researchers have also reported a remarkable increase in the TPC of biopolymer‐based films due to the incorporation of PPP and pomegranate peel extract (Kumar et al., 2021).

### 3.4 Antioxidant activity of cassava starch films containing PPP

**FIGURE 6:**
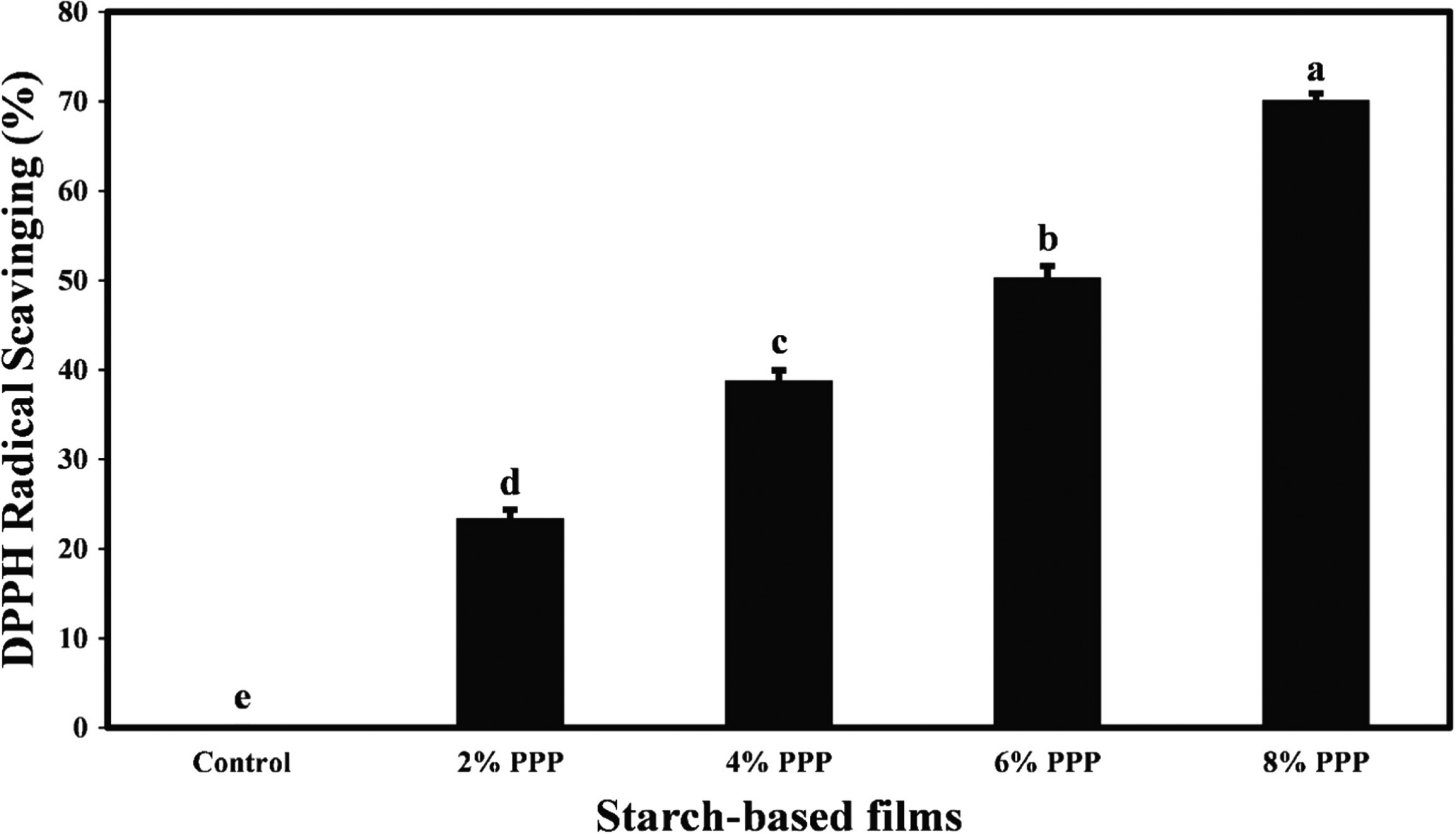
Comparison of DPPH radical scavenging means values (%) of cassava starch‐based films containing different levels of pomegranate peel powder. Bars represent mean (*n* = 3) ± *standard deviation*. Different letters on the bars indicate a significant difference at 5% level of probability among film samples. DPPH: 1,1‐diphenyl‐2‐picryl hydrazyl. PPP: pomegranate peel powder

Free radicals can reduce the nutritional quality, safety, and spoilage of packaged food products. Therefore, free radical‐scavenging and antioxidant activity are major characteristics of films used for food packaging (Wang et al., 2013). The antioxidant activity of cassava starch films containing different levels of PPP was evaluated by DPPH free radical‐scavenging ability assay, and the results are demonstrated in Figure 6. As shown in the figure, since cassava starch film without additive (control) did not contain phenolic and functional compounds, it did not show any antioxidant activity. By adding PPP to the starch‐based film formulation and increasing its levels in the samples, DPPH free radical scavenging was significantly increased (*p* < .05), and the highest antioxidant activity (70.14%) was observed in the film containing the highest concentration of PPP (8% level). In general, the presence of remarkable amounts of various phenolic compounds such as anthocyanins (delphinidin, cyaniding, 3,5‐diglucosides, and pelargonidin‐3‐glucosides), gallotannins, hydrolyzable tannins, hydroxybenzoic acids, hydroxycinnamic acids, etc., in pomegranate peel has been confirmed, which leads to strong antioxidant activity in it (Akhtar et al., 2015).

Mabrouk et al. (2019) and Maroufi et al. (2021) achieved similar results in investigating pomegranate peel's effect on pectin‐based edible films’ antioxidant activity of chitosan‐modified dialdehyde guar gum films, respectively. The addition of lemon peel powder to polyvinyl alcohol–starch films also significantly increased the antioxidant activity of the film samples (Terzioglu & Sicak, 2021).

### 3.5 Study of the relationship between TVB‐N of lamb meat and the color changes of cassava starch film containing PPP

**FIGURE 7:**
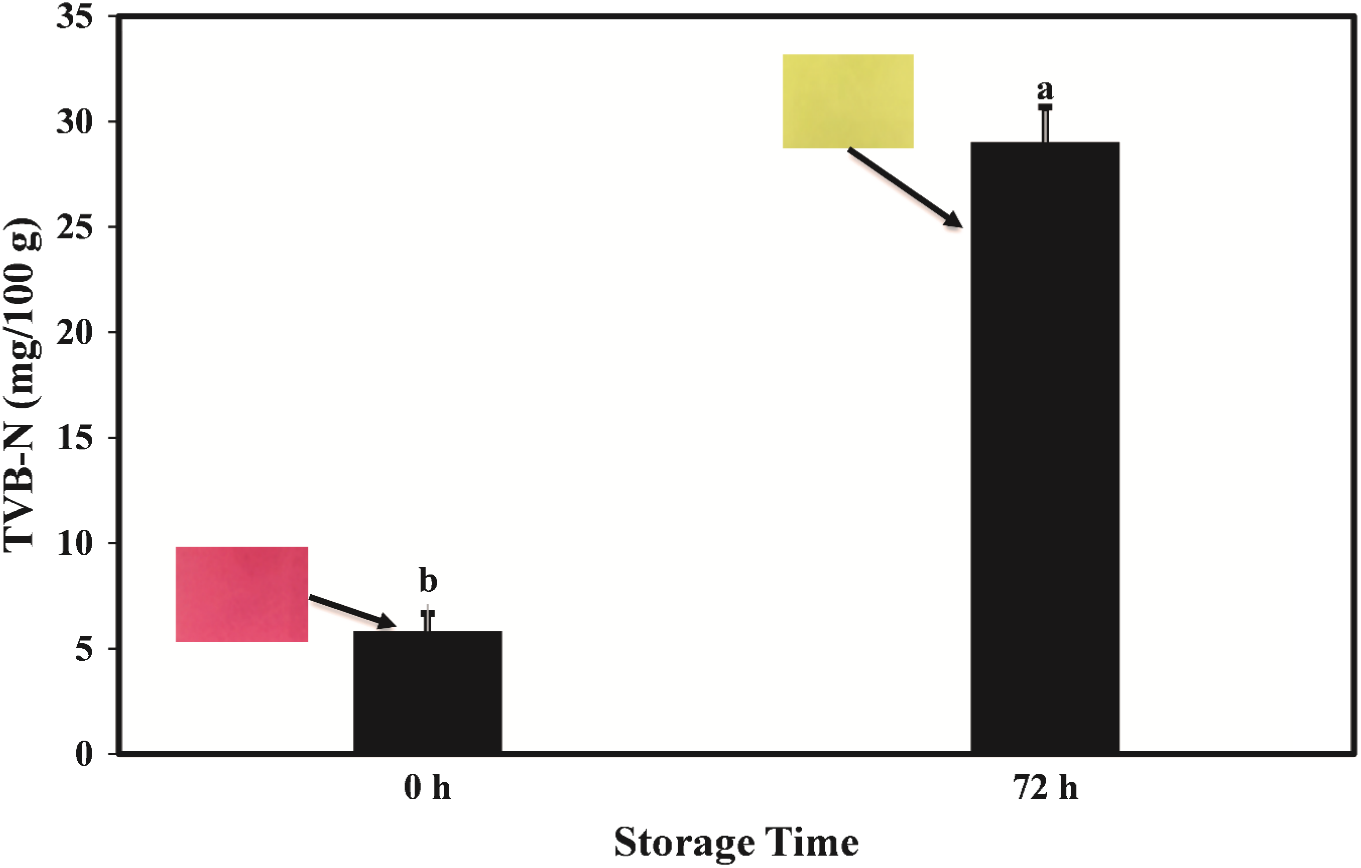
The relationship between the color of cassava starch‐based films containing pomegranate peel powder and the TVB‐N value of lamb meat. PPP: pomegranate peel powder. TVB‐N: total volatile basic nitrogen. The color of cassava starch smart films changed from purple to greenish yellow by increasing TVB‐N

In general, most spoilage of protein‐based food products occurs due to the growth of microorganisms. Degradation of protein structures by spoilage microorganisms leads to high levels of TVB‐N, including dimethylamine, trimethylamine, and ammonia, which can change the pH of the interface (Liu et al., 2018). The maximum acceptable level for TVB‐N in fresh meat and meat products is 30 mg/100 g. In this study, an active and intelligent cassava starch‐based film containing 8% PPP (as a rich source of anthocyanins) was used to evaluate the efficiency of showing the freshness of lamb meat at 25°C, and the results are presented in Figure 7. As the figure shows, the amount of meat TVB‐N at the beginning of the experiment was 5.82 mg/100 g, and after 72 h of storage at 25°C, the amount of TVB‐N remarkably increased to 29.03 mg/100 g. The experimental results demonstrated that the color of the film changed from purple to green. Therefore, these results showed that cassava starch‐based film containing PPP could be used effectively to monitor the freshness of lamb meat during the storage period.

Generally, various factors affect the color of anthocyanins, including temperature, oxygen presence, pH, enzymatic decomposition, the chemical structure of anthocyanin, and reaction with other compounds such as sugars, pigments, and metal ions (Patras et al., 2010). Anthocyanins are purple and red at acidic pHs (pH = 1–3). At pH 6, the flavylium cation concentration decreases and colorless carbinol pseudobase and chalcone are produced by the hydration process. At pH 7, purple quinoidal anhydrobase, and pH above 8, deep blue ionized anhydrobase is formed. At more alkaline pHs, a yellow or green chalcone is produced by opening the central pyran ring of carbinol (Wardana & Widyaningsih, 2017; Yoshida et al., 2014).

Similarly, Chi et al. (2020) examined the relationship between the TVB‐N of pork meat and color change of kappa‐carrageenan‐based films containing red grape peel. They found that during storage at room temperature, the amount of TVB‐N increased significantly and when TVB‐N reached the maximum recommended amount, the color of the films changed from pink to green. In the research conducted by Liu et al. (2018), a kappa‐carrageenan‐based film containing curcumin was used to display the freshness of pork and shrimp. The results showed that on the third day of storage, the color of the intelligent film changed from yellow to red, which was related to the change in the TVB‐N values of the studied meats. Zhang et al. (2022) also observed that the color of ovalbumin–propylene glycol alginate films containing anthocyanins changed from purplish‐red to dark blue as the freshness of pork decreased during storage. The change in the color of starch–carbon dot films containing anthocyanin (extracted from *Clitoria ternatea* flower) from purple to green has also been reported by Koshy et al. (2021).

## 4 CONCLUSION

This study demonstrated that the addition of PPP had a remarkable effect on the TPC and antioxidant activity of cassava starch‐based films. A direct relationship was observed between the TPC of the active films and their antioxidant activity. Active films had a darker, redder, and yellower color than control films. There was also a relationship between the color change of the intelligent film and the TVB‐N content of lamb meat. When TVB‐N value was acceptable, the film was reddish‐purple, but when the amount of meat TVB‐N reached the maximum acceptable level, the film color turned green. Finally, the results of this study indicated the potential of cassava starch‐based film containing PPP as a pH‐sensitive intelligent film to monitor the freshness of meat.

## CONFLICT OF INTEREST

The authors declare no conflict of interest.

## ETHICAL APPROVAL

This study does not involve any human or animal testing.

## Data Availability

The data that support the findings of this study are available from the corresponding author upon reasonable request.
